# Germline polymorphisms as modulators of cancer phenotypes

**DOI:** 10.1186/1741-7015-6-27

**Published:** 2008-09-08

**Authors:** Patrick Tan

**Affiliations:** 1 Duke-NUS Graduate Medical School, 2 Jalan Bukit Merah, 169547, Singapore; 2 Genome Institute of Singapore, 60 Biopolis Street, 138672, Singapore

## Abstract

Identifying the complete repertoire of genes and genetic variants that regulate the pathogenesis and progression of human disease is a central goal of post-genomic biomedical research. In cancer, recent studies have shown that genome-wide association studies can be successfully used to identify germline polymorphisms associated with an individual's susceptibility to malignancy. In parallel to these reports, substantial work has also shown that patterns of somatic alterations in human tumors can be successfully employed to predict disease prognosis and treatment response. A paper by Van Ness et al. published this month in *BMC Medicine *reports the initial results of a multi-institutional consortium for multiple myeloma designed to evaluate the role of germline polymorphisms in influencing multiple myeloma clinical outcome. Applying a custom-designed single nucleotide polymorphism microarray to two separate patient cohorts, the investigators successfully identified specific combinations of germline polymorphisms significantly associated with early clinical relapse. These results raise the exciting possibility that besides somatically acquired alterations, germline genetic background may also exert an important influence on cancer patient prognosis and outcome. Future 'personalized medicine' strategies for cancer may thus require incorporating genomic information from both tumor cells and the non-malignant patient genome.

## Commentary

### Germline variations and human health

A major advancement of genetic research in recent years has been the explosion of genome-wide association studies (GWAS) in the literature from different investigators and laboratories [1]. The completion of the reference human genome sequence, and its subsequent comparison across different human sub-populations, has identified millions of genetic polymorphisms that differ between different individuals, families, and ethnic groups [2]. With the availability of increasingly affordable chip technologies for interrogating these polymorphisms *en masse *in individual genomes, it is now possible to consider identifying, on a comprehensive genome-wide scale, all genes and genetic variants associated with human disease. In the area of cancer, GWAS studies have been performed for multiple different tumor types including breast, lung, and stomach cancers [3-5]. These studies have both reconfirmed previously known disease genes (eg *FGFR2 *in breast cancer) [3], and also identified novel genetic loci, such as *TNRC9*, *MAP3K1*, and *LSP1 *for breast cancer [3] and the nicotinic acetylcholine receptor subunits in lung cancer [4]. To date, the majority of reported GWAS studies have employed a case-control design, where affected individuals with a disease are compared against a matched population of non-affected normal controls. The genetic variants identified using such case-control designs thus represent 'disease susceptibility' loci that can either increase or decrease an individual's risk to developing disease. A report published this month in *BMC Medicine *by Van Ness et al. [6] seeks to extend this theme, by asking whether germline polymorphisms can influence not simply the onset of disease, but the actual course of disease prognosis in cancer.

### Somatic alterations as dominant drivers of cancer progression

The focus of Van Ness et al. on the cancer patient germline is particularly notable when one considers how the concept of cancer as an acquired somatic disease has dominated the field. In this model, tumor cells are believed to arise as a consequence of accumulated genetic lesions, which cause the pathologic activation of oncogenes and inactivation of key tumor suppressor pathways [7]. Furthermore, multiple studies have already described various somatically-derived genomic 'signatures' in tumors that can predict both disease outcome and response to therapy, such as a 70 gene expression signature in breast tumors that can identify patients with particularly good prognoses [8], and *EGFR *mutations in lung cancers that can predict tumor response to *EGFR*-targeted therapies [9,10]. In contrast to the altered cancer genome, studies analyzing the germline analysis of cancer patients, for the most part, have been largely confined to the pharmacodynamic/pharmacokinetic (PK/PD) arena, where patients are genotyped for polymorphisms in various drug-metabolizing genes to identify individuals at greatest risk of incurring severe drug toxicities (eg *UGT1A1 *in irinotecan treatment) [11].

More recently, however, emerging evidence suggests that in addition to somatic alterations, germline variations may also play an important role in influencing cancer prognosis and disease outcome. This might occur if particular germline variants increase the risk of developing a particular cancer subtype intrinsically associated with poor prognosis. For example, in stomach cancer, polymorphisms in the *PSCA *gene have been shown to be associated with the development of diffuse-type gastric adenocarcinoma, a histologic variant traditionally associated with poor clinical outcome [5]. The influence of host genetic background on the development of cancers with differing metastatic traits has also been observed in mouse models of cancer [12]. Germline polymorphisms could also influence cancer prognosis by affecting the regulatory circuitry of cancer cells, by altering promoter-binding sites for important cancer-related genes such as *mdm2*, a negative regulator of *p53 *[13]. Although such studies are still relatively few in number, they do suggest that it may be time to initiate more systematic efforts to understand the specific role of germline genetic background in determining the course of cancer progression.

### Germline variants may affect outcome in multiple myeloma

The report by Van Ness et al. provides promising initial data that this idea may indeed have scientific merit. This group has focused on multiple myeloma (MM), a hematopoetic malignancy of plasma B-cells. Although considered a uniformly fatal disease, individual MM patients are known to exhibit significant clinical heterogeneity in terms of disease morphology, time to progression and response to treatment [14]. To ask whether germline polymorphisms might underlie some aspect of this clinical heterogeneity, the investigators designed a customized single nucleotide polymorphism (SNP) array to measure genetic polymorphisms across ~1000 genes in biological pathways relevant to MM or MM therapy, including immunity and inflammatory pathways, and genes related to drug metabolism and transport. Although not a genome-wide approach, the use of a targeted array is not without its advantages. First, by making use of prior literature knowledge, the investigators were able to incorporate many SNPs in pathways and genes relevant for MM not represented on standard genome-wide SNP arrays. Second, the use of a smaller SNP set (3500 SNPs) allowed the study to be performed with relatively smaller numbers of patient samples while still preserving statistical power, compared with a typical GWAS study. Third, because the choice of treatment regimen is an important contributor to clinical outcome, the use of smaller numbers of patient samples also facilitates standardization of treatment therapies across independent patient cohorts.

The investigators applied their customized array to two separate cohorts of MM patients treated with comparable chemotherapeutic regimens. The recruitment of these patients was orchestrated through 'Bank on a Cure' (BOAC), a centralized collection agency for MM patient material from different corporative groups and institutional trials, established by expert researchers and clinicians in the MM field [15]. Using a series of computational training algorithms, the investigators showed that they could classify the patients on the basis of the germline SNP profiles into two distinct groups of 'good prognosis' (>3 year progression-free survival, PFS) vs 'bad prognosis' (<1 year PFS) groups above random chance. Although this initial result will undoubtedly require further validation to assess its ultimate accuracy, several intriguing trends have already emerged from their data. Among these, the authors found that accurate classification was highly dependent on using a multiplex panel of SNPs rather than any single SNP in isolation, strongly suggesting that the factors driving disease outcome in MM are likely to be complex and multifactorial. Another interesting finding was that patient classification accuracy also increased when the analysis was restricted to non-synomymous SNPs, ie those SNPs causing amino acid differences in proteins. This may imply that stronger effects on clinical outcome are likely to be modulated through alterations in protein function, rather than by alterations in the regulatory pathways controlling the transcription of these genes.

### Challenges for the future

Although obviously exploratory in nature, the promising results of this initial study have paved the way for more ambitious and rigorous experimental designs and projects. Some potential issues with the current study include the somewhat arbitrary threshold for defining the prognosis categories, which focuses on extreme cases (<1 year vs >3 years). It would have been interesting, for example, to ask if similar SNP associations were also observed if the analysis was repeated treating patient survival as a continuous variable. It could also be argued that the lack of using an unbiased genome-wide approach prohibited the investigators from discovering potentially novel gene-disease associations, which might have shed further light on the regulatory pathways underlying MM. Ultimately, because this approach is relatively new, the study data will have to be further scrutinized in several other independent cohorts to assess the true prognostic power of these SNPs.

**Figure 1:**
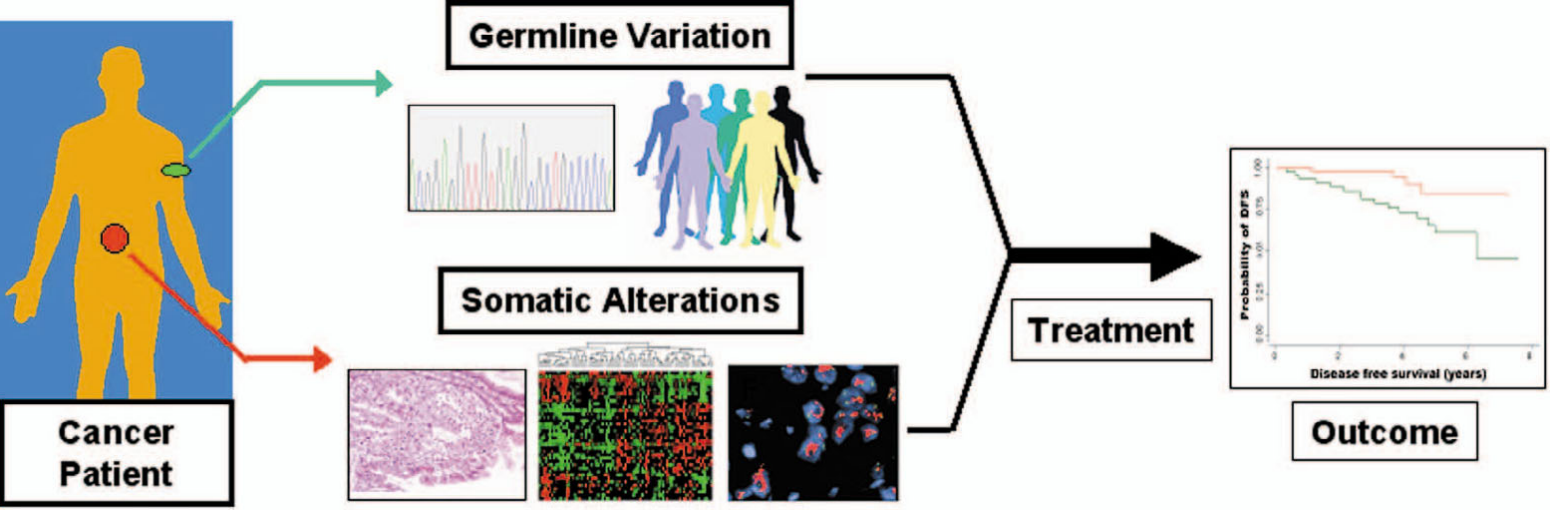
**Determinants of clinical outcome in cancer**. The schematic outlines the interaction between germline genetic background and somatic alterations in influencing cancer outcome. Germline variations may result in differences between individuals in drug metabolizing activities, cancer pathways, and development of distinct molecular subtypes of cancer (top boxes). Alternatively, somatic alterations can cause differences in histopathology, gene expression, and gene amplifications and deletions (bottom boxes). Overlaid upon this germline/somatic interaction is the specific choice of treatment regimen. All these factors interplay to ultimately determine patient outcome (Kaplan-Meier survival curve on right). Pictures of human figures were adapted from . Pictures of FISH images and Kaplan-Meier survival curves were generated in the author's laboratory and previously used in [18].

Finally, it will be fascinating to determine whether the stratification power provided by these germline SNPs reflects an enhanced propensity of certain patients to develop a particular poor or good prognosis 'molecular subtype' of MM, since there is already a significant body of work describing various somatic alterations in MM that are predictive of clinical outcome [16,17]. Alternatively, it is also possible that these survival-associated SNPs may provide further stratification power beyond that observed by studying the somatic MM genome alone. In conclusion, this study by Van Ness is quite exciting and likely represents a model for similar studies in other cancers. It recognizes that the factors determining disease outcome in cancer are complex and multifactorial, ranging from the propensity of a cancer to proliferate and metastasize to how that cancer might respond to different types of therapy. One possibility for the future might be to test how such germline information can best be further integrated with somatic genomics to derive a holistic model for predicting outcome (Figure 1). Such information might directly translate to the development of new pharmaceutics, and diagnostic panels for personalized and predictive medicine.

## Pre-publication history

The pre-publication history for this paper can be accessed here:


